# 

**Published:** 1891-01

**Authors:** 


### CONTENTS.

ORIGINAL ARTICLES—Hypertrophy of the Tonsils—By T. Hilliard Wood, M. D., Nashville, Tenn .... 1
Fracture of Rib, Followed by Extensive Tissue Emphysema—By C. D. Roy, M. D., New York.... 7
Fracture at Elbow Joint, with Cases—By Andrew Boyd, M. D., Scottsboro, Ala.... 12
Physiological Action of Chloroform—By E. Van Goidtsnoven, M. D., Atlanta, Ga.... 17
CORRESPONDENCE—Our New York Letter.... 20
SOCIETY NOTES—Proceedings of the Richmond (Va.) Academy of Medicine and Surgery—A Case of Laparotomy—Osteo-Sarcoma of Femur, Amputation—Triplets.... 24
BOOK REVIEWS—Comparative Physiology—By Wesley Mills, M. D. The Physician's Visiting List—By P. Blakiston, Son & Co. Saunders' Pocket Medical Lexicon—By J. M. Keating, M. D., and Henry Hamilton. Ointments and Oleates—By J. V. Shoemaker, M. D. A Manual of Auscultation and Percussion—By Austin Flint, M. D. The Medical Bulletin Visiting list for 1891. The Medical News Visiting List for 1891. Weekly Medical Review Visiting List. Medical Diagnosis with Special Reference to Practical Medicine—By J. M. Da Costa, M. D.... 29-34
EDITORIAL—Another Appeal—Atlanta Society of Medicine.... 35-36
SELECTIONS AND ABSTRACTS—Notes upon Somnal, the New Hypnotic—Mosquera's Food Products, Beef Meal, Beef Cacao—Gonorrhoeal Rheumatism—The Rational Treatment of Pneumonia—Chapped Lips—The Industrial Future of the South—Cardiac Hypertrophy—Vomiting in Pregnancy—Swallowed a Gold Safety-Pin—Infantile Convulsions, etc.... 37
LA GRIPPE—Its Treatment in Our City—By Drs. J. A. Childs, Wm. Perrin Nicolson, N. O. Harris, L. H. Jones, L. B. Grandy, J. C. Johnson, F. O. Stockton, W. C. Jarnagin, C. G. Giddings, and J. W. Duncan.... 49-54

